# Modeling the Effect of Primary and Secondary Twinning on Texture Evolution during Severe Plastic Deformation of a Twinning-Induced Plasticity Steel

**DOI:** 10.3390/ma11050863

**Published:** 2018-05-22

**Authors:** Laszlo S. Toth, Christian Haase, Robert Allen, Rimma Lapovok, Dmitri A. Molodov, Mohammed Cherkaoui, Haitham El Kadiri

**Affiliations:** 1 Laboratoire d’Etude des Microstructures et de Mécanique des Matériaux (LEM3), Université de Lorraine, CNRS, Arts et Métiers ParisTech, LEM3, 57000 Metz, France; laszlo.toth@univ-lorraine.fr; 2 Laboratory of Excellence on Design of Alloy Metals for Low-mAss Structures (DAMAS), Université de Lorraine, 57045 Metz, France; 3 Steel Institute, RWTH Aachen University, 52072 Aachen, Germany; 4 Department of Mechanical Engineering, Mississippi State University, Starkville, MS 39762, USA; rma140@msstate.edu (R.A.); cherkaou@cavs.msstate.edu (M.C.); elkadiri@me.msstate.edu (H.E.K.); 5 Institute for Frontier Materials, Deakin University, Geelong, Victoria 3217, Australia; r.lapovok@deakin.edu.au; 6 Institute of Physical Metallurgy and Metal Physics, RWTH Aachen University, 52074 Aachen, Germany; molodov@imm.rwth-aachen.de

**Keywords:** TWIP steel, ECAP, deformation twinning, texture, VPSC, simulation

## Abstract

Modeling the effect of deformation twinning and the ensuing twin-twin- and slip-twin-induced hardening is a long-standing problem in computational mechanical metallurgy of materials that deform by both slip and twinning. In this work, we address this effect using the twin volume transfer method, which obviates the need of any cumbersome criterion for twin variant selection. Additionally, this method is capable of capturing, at the same time, secondary or double twinning, which is particularly important for modeling in large strain regimes. We validate our modeling methodology by simulating the behavior of an Fe-23Mn-1.5Al-0.3C twinning-induced plasticity (TWIP) steel under large strain conditions, experimentally achieved in this work through equal-channel angular pressing (ECAP) for up to two passes in a 90° die following route B_C_ at 300 °C. Each possible twin variant, whether nucleating inside the parent grain or inside a potential primary twin variant was predefined in the initial list of orientations as possible grain of the polycrystal with zero initial volume fraction. A novelty of our approach is to take into account the loss of coherency of the twins with their parent matrix under large strains, obstructing progressively their further growth. This effect has been captured by attenuating growth rates of twins as a function of their rotation away from their perfect twin orientation, dubbed here as “disorientation” with respect to the mother grain’s lattice. The simulated textures and the hardening under tensile strain showed very good agreement with experimental characterization and mechanical testing results. Furthermore, upper-bound Taylor deformation was found to be operational for the TWIP steel deformation when all the above ingredients of twinning are captured, indicating that self-consistent schemes can be bypassed.

## 1. Introduction

High-manganese twinning-induced plasticity (TWIP) steels are promising candidates for application in crash-relevant automobile components due to their outstanding mechanical properties [1,2]. These properties, i.e., high strength, ductility, and work-hardening capacity, originate from their low stacking fault energy (SFE), which is typically in the range between ~20 mJ/m^2^ and ~50 mJ/m^2^ and enables the activation of deformation-induced twinning in addition to planar dislocation slip, while strongly impeding cross-slip [3]. The high work-hardening rates that are responsible for the excellent deformation behavior are attributed to the combination of strong planarity of dislocation motion and the dynamic Hall-Petch effect that facilitates drastic reduction of the dislocations’ mean free paths due to the formation of nano-scale deformation twins [4,5].

Besides their effect on the mechanical properties, the deformation mechanisms active in high-manganese steels have a strong influence on texture evolution. So far, this has been analyzed mainly during cold rolling [6,7,8,9,10,11,12,13], recrystallization [7,14,15,16,17], and tensile testing [18,19,20,21]. In contrast to the conventional processing of TWIP steels, severe plastic deformation of these alloys has only been sparsely investigated, even though it was found to have a significant effect on their microstructural evolution and mechanical properties [22,23,24,25,26]. In particular, the understanding of the correlation between deformation mechanisms and texture evolution is limited. In our recent study [26], the equal-channel angular pressing (ECAP) method was used to deform an Fe-23Mn-1.5Al-0.3C TWIP steel up to four passes at 300 °C following route B_C_. Experimental analysis revealed that the microstructure during ECAP was continuously refined, owing to both grain subdivision via deformation twinning and by dislocation-driven grain fragmentation. Due to the increased SFE at 300 °C, the latter mechanism dominated the microstructure evolution. In accordance with the correspondence introduced by Suwas et al. [27,28], a transition texture is formed that contains texture components characteristic of materials with both high and low SFE. Shear bands, if they occur, appear parallel to the applied shear in shear deformation [29]. As they were hardly observed during ECAP of TWIP steel [26], they were not considered in the present modeling work.

Previously developed methods for modeling the evolution of twin volume fractions and their effects on texture and mechanical hardening begin with the pioneering work of Van Houtte [30], which utilized a statistically based criterion for the selection of parent grains to be reoriented into twin orientations. Later works by Tomé et al. [31] and Lebensohn and Tomé [32] introduced the predominant twin reorientation (PTR) scheme, in which the mother grain is replaced completely by its most active twinning variant once its activity surpasses a critical value. Implemented as part of the viscoplastic self-consistent code, VPSC-7d, the PTR scheme was further developed in order to explore the relationship between twinning and hardening evolution [32,33,34]. However, both methods were limited in that neither allowed for the direct representation of the effects of multiple twin systems on the evolution of texture in simulated polycrystals. Other methods have been developed in order to address this limitation. The volume fraction transfer (VFT) scheme of Tomé et al. [31] calculated changes in texture by discretizing Euler space into separate orientation cells whose centers are occupied by the orientations of physical grains. As the grains are reoriented either continuously through rotations encountered by slip, or discontinuously in the case of twinning, the cells in the orientation space associated with these grains are shifted via translation vectors or by specific vector transformations for twinning, respectively, in Euler space. The volume fraction of each grain is then modified by an amount proportional to the overlap of the newly positioned Euler cells. The volume transfer approach of Kalidindi [33] introduces each twinning variant as a physical grain from the beginning, and the grain-weights are modified according to the twin activity: volume fractions are transferred from the mother grain to each of the twin-variant as a function of the pseudo-slip that a twin contributes to the deformation of the mother grain. This technique was adopted for large deformation simulations in recent works of Toth and co-workers to reveal the effect of nano-twins on texture evolution for rolling of ultra-fine-grained copper [34,35] and for twinning-detwinning activity in the fatigue of Mg AZ31 [36]. Twinning in TWIP steel was modelled by Prakash et al. [37], who created only one twin-variant as a new grain, which was selected by the PTR scheme among the 12 possible ones. The full volume transfer scheme for twinning in TWIP steel is applied for the first time in the present work, and without the use of the PTR scheme.

In this work, we aim to shed more light on the influence of deformation twinning on the texture evolution of face-centered cubic (fcc) alloys with low SFE, such as the high-manganese steel investigated here. To model the texture evolution during ECAP, an extended version of the viscoplastic self-consistent (VPSC) polycrystal code, developed in Metz, was used [38,39]. Nevertheless, the Taylor version of the VPSC model was identified to be applicable for the case of heavy large strain deformation twinning in TWIP steel. In addition to dislocation slip, primary and secondary twinning were implemented in the simulation code by predefining all possible twin variants. The volume fractions of twins were increased according to the activity of the twin systems, which were considered pseudo-slips in their parent grains, without variant selection. One of the main aims of the study is to examine the question as to whether the texture evolution can be simulated without applying any twin variant selection criteria. Another main novelty of our approach is to take into account the loss of twin orientation relationship between the twin and the mother grain in the growth process of the twins. This loss occurs due to the different lattice rotations of the twin and the matrix during the very large strains that are imposed. Finally, the different hardening characteristics of the twins with respect to the matrix are taken into account by a sophisticated self and latent hardening model. The obtained results show that our modeling provides a genuine approach for a quantitative simulation of the deformation behavior of TWIP steels.

## 2. Material and Methods

The chemical composition of the investigated TWIP steel is given in Table 1. The SFEs at room temperature and at 300 °C were calculated to be ~25 mJ/m^2^ and ~75 mJ/m^2^, respectively, using a subregular solution thermodynamic model [40].

**Figure 1:**
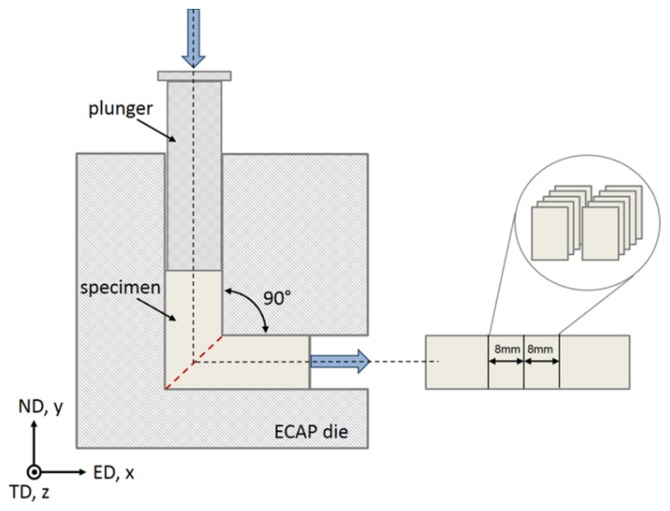
Schematic diagram of the reference coordinate system and sample taking according to the die geometry. ED, ND, and TD denote extrusion, normal, and transverse direction, respectively.

After casting, homogenization, annealing, forging, and hot rolling, rods with a diameter of 10 mm and a length of 35 mm were cut out perpendicular to the rolling direction for further deformation by ECAP. The ECAP rig used for the experiments is described in detail in [41]. The ECAP experiments were performed at 300 °C following route B_C_ up to two passes using a 90° ECAP die at a pressing rate of 1 mm/s. The chosen value of the pressing speed is based on previous studies [42]. The die geometry and the reference system for the testing is given in Figure 1. In the following, the ED, ND, and TD directions mean extrusion, normal, and transverse directions, respectively. Specimens for microstructure and texture characterization were cut from the deformed rods with dimensions of 8 mm (ED) × 10 mm (ND) × 1 mm (TD). Sample preparation on the ED-ND sections consisted of mechanical grinding and polishing, followed by electro-polishing at room temperature and 22 V using an electrolyte containing 700 mL ethanol (C_2_H_5_OH), 100 mL butyl glycol (C_6_H_14_O_2_), and 78 mL perchloric acid (60%) (HClO_4_). Transmission electron microscopy (TEM) samples (~100 µm initial thickness, 3 mm in diameter) were prepared using the same electrolyte in a double-jet Tenupol-5 electrolytic polisher with a voltage of 25 V at room temperature.

Electron backscatter diffraction (EBSD) measurements were performed using a LEO 1530 field emission gun scanning electron microscope (FEG-SEM) (Carl Zeiss AG, Oberkochen, Germany) operated at a 20 kV accelerating voltage and a working distance of 10 mm. EBSD maps were acquired applying a step size of 0.15 µm and were post-processed utilizing the HKL Channel 5 software (Oxford Instruments plc, Abington, Great Britain). The TEM specimens were analyzed using a JEOL JEM 2000 FX II analytical TEM (JEOL Ltd., Akishima, Japan) operated at 200 kV.

In order to characterize the crystallographic textures, X-ray pole figure measurements were performed by acquiring three incomplete (0–85°) pole figures {111}, {200}, and {220}. A Bruker D8 Advance diffractometer (Bruker Corporation, Billerica, USA), equipped with a HI-STAR area detector, operating at 30 kV and 25 mA, using filtered iron radiation and polycapillary focusing optics was used. The corresponding orientation distribution functions (ODFs) were calculated using the JTEX software (Universite de Lorraine, Metz, France) [43].

## 3. Experimental Results

The main experimental findings were presented in a previous study [26]. It has been found that ECAP of the investigated TWIP steel at 300 °C led to the formation of a shear texture, with texture components that are characteristic for both low- and high-SFE materials. Therefore, the texture was described as a transition texture, based on the correspondence suggested by Suwas et al. [27,28]. The accommodation of plastic strain by both dislocation driven grain fragmentation and deformation twinning were found to be the reason for the formation of this transition texture.

In the following, the experimental results that serve as basis for the present modeling approach are described briefly.

**Figure 2:**
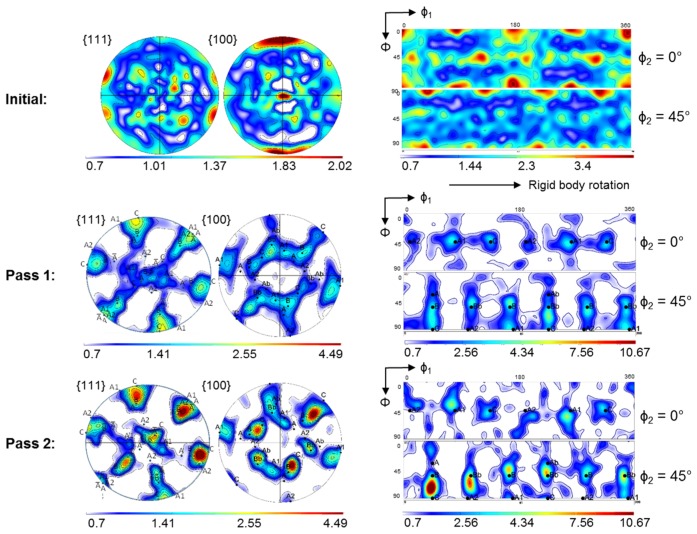
Experimental textures in {111} and {100} pole figures as well as in the φ_2_ = 0° (**top**) and 45° (**bottom**) ODF sections. Reference system for the pole figures: vertical axis (*x*) is the outgoing channel axis direction, horizontal axis (*y*) is opposite to the pressing direction, center (*z*) is the transverse direction. The Euler angles of the ODFs refer to the same (*x*, *y*, *z*) reference system. The range of φ_1_ angle (horizontal axis) is from 0° to 180°, the Φ is from 0° to 90°, in top to bottom direction.

The initial texture was relatively weak (Figure 2). The strongest component was the {001}<100> cube texture component, with an intensity of about 2.0 in the {100} pole figure. After one ECAP pass, a shear texture appeared, with relatively strong A1, C, and B/Bb components. The A2 and A/Ab components were relatively weak. For the characteristics of these ideal components, see Table 2 and Ref. [44,45]. (Note that Bb means the $\bar{B}$ component; similarly, Ab is $\bar{A}$ in previous publications). The texture was comparatively weak with a texture index of 2.14 and the ideal components were generally surrounded by other, weaker texture components.

After two passes (Figure 2), the ODF maximum was located between the C and B components, with higher intensity than the peaks after the first pass (texture index: 2.8). The total surface area of low intensities in the two ODF sections was reduced compared to that after the first pass.

**Figure 3:**
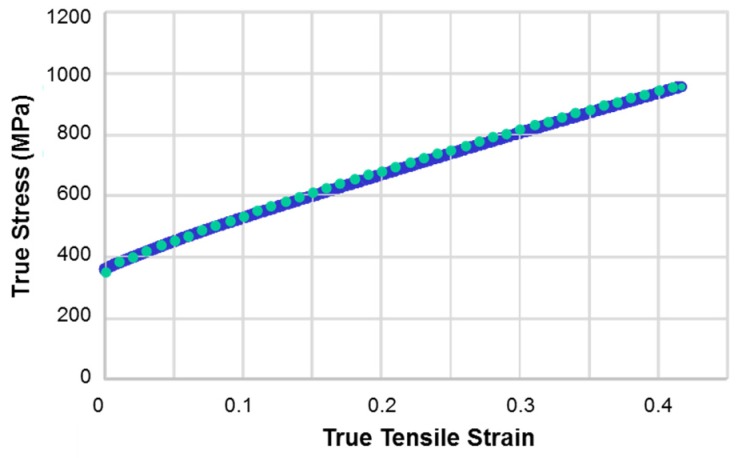
Tensile stress-strain curve of the TWIP steel in initial condition before ECAP, tested in RD of hot rolling. Continuous line: experiment, dotted line: simulation. The experiment was performed at a strain rate of 10^−3^ s^−1^ at room temperature.

The true stress-true strain curve obtained from tensile testing of the investigated steel in its initial hot-rolled condition before ECAP is displayed in Figure 3. As can be seen, the strain hardening was very substantial and the curve increases nearly linear.

**Figure 4:**
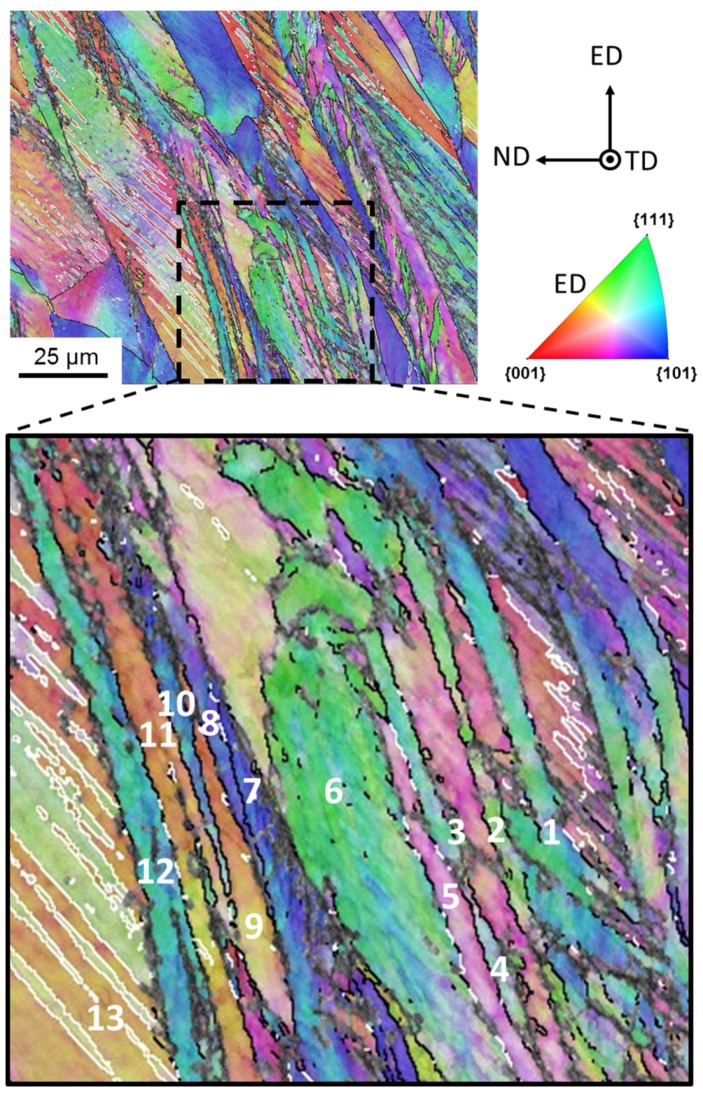
EBSD map with color-coding according to the inverse pole figure after one-pass ECAP. 13 grains were analyzed in the selected zone as illustrated in the inset. Black and white lines denote high-angle grain boundaries (*θ* ≥ 15°) and ∑3 (60°<111>) grain boundaries, respectively.

**Figure 5:**
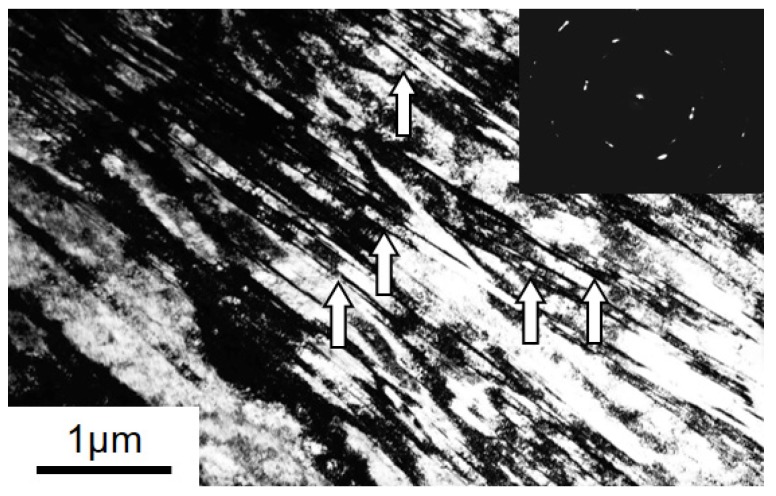
Bright field TEM micrograph and the corresponding selected area diffraction pattern after one ECAP pass showing a high fraction of deformation twins. Selected twins/twin-matrix lamellae are indicated by arrows.

As expected for TWIP steel, deformation twinning was pronounced in the studied material during ECAP. Figure 4 shows that the elongated twin lamellae were abundant in the deformation microstructure. Finer details of the microstructure could be seen by TEM, see Figure 5. Very fine twin lamellae were found with thicknesses in the nanometer range, between 40 nm and 100 nm (Figure 5). While, at their creation, the twins must appear in perfect twin-orientation relationship with respect to the parent grain, it is not guaranteed that the ideal orientation relationship will be maintained during plastic deformation up to large strains. In order to verify the loss of orientation relationship between parent grain and its twin, an analysis was carried out on the EBSD-observed microstructure. The inset in Figure 4 shows the analyzed region in the EBSD map after one ECAP pass. Some selected adjacent grains labeled in Figure 4 with 1 to 13 were examined in terms of their misorientation. The mean Euler angles of these 13 grains are given in Table 3. 

Table 4 contains the results of the twin-orientation analysis, i.e., the disorientation angle of each pair of neighboring grains together with their nearest disorientation axis, the offset from the nearest rotation axis, and the deviation angle from the ideal 60°<111> twinning orientation relationship. The deviations were typically around 10°, but there were also several larger disorientations, up to 50°. This analysis provided evidence that the twins drifted away from their initial ideal orientation relation with respect to their parent grains during deformation, i.e., they did not co-rotate with the parent grain after large strains.

## 4. Modeling Approach

### 4.1. The Polycrystal Model

In this work, simulation of the texture evolution was performed using the VPSC polycrystal code developed in Metz, in its finite-element tuned version [38,39]. The code itself is considered an ‘isotropic’ code, so-called because the interaction between a given grain and the homogeneous medium is calculated using the assumption that the medium itself is isotropic. Although not appropriate for hexagonal materials, this assumption can be considered valid for cubic materials without strong textures. As TWIP steels possess an fcc crystal structure and usually show weak textures [46], such conditions are expected to be met. The volume transfer scheme recently introduced in publications by Toth et al. to account for twinning [35,36] was implemented in the VPSC code; simultaneously for both primary and secondary twinning.

### 4.2. Twinning Approach

In the volume transfer scheme, each twin variant is considered as a separate grain created at the beginning of the simulation with zero initial volume fraction. As deformation progresses during the simulation, volume fraction (Δv)^twin^ is transferred from the parent grain to its corresponding twins. This approach can only be applied if the twin-parent orientation relation does not deviate too much from the ideal one. A certain deviation angle can be permitted because the local dislocation mechanisms that make the twin grow can accommodate a small difference. In the present work, the volume attributed to the twin from the parent grain was reduced progressively as a function of the deviation of the orientation of the twin from its exact twinning position. After each strain increment Δt, the disorientation angle (*θ*) between the ideal twin position and the actual orientation of the twin was calculated. Then the <112> slip system activity was examined in order to transfer some part of the volume of the parent grain to the twins. The volume was calculated in proportion to the <112> slip increment normalized by the shear necessary to form a twin ($\gamma_{\mathrm{twin}}=1/\sqrt{2}$). The deviation from the exact twin position was also taken into account in the amount of volume transfer:

(1) $${\left(\Delta v\right)}^{\mathrm{twin}}=\frac{\left(10{}^{\circ}-\theta \right)}{10{}^{\circ}}\frac{\left(\Delta t\times {\dot{\gamma }}^{\mathrm{twin}}\right)}{\gamma_{\mathrm{twin}}}V_{\mathrm{parent}},\;\mathrm{for}\;\theta \leq 10{}^{\circ},\;{\left(\Delta v\right)}^{\mathrm{twin}}=0,\;\mathrm{for}\;\theta >10{}^{\circ}.$$

When the deviation angle exceeded 10°, the volume transfer was stopped. Once formed, the twins continued deforming using their available slip systems. The <112> type slip activity was used for growing the twin; however, they did not grow any more once they rotated out of their ideal twinning position with respect to their parent grains.

In order to avoid artifacts related to twins with extremely small volume fractions, the volume transfer from the mother grain to a twin variant was only initiated if the mother grain was slipping on the corresponding <112> slip at least by 1% of the total slip of the mother grain. (Here, the “corresponding <112> slip” means the twin variant that corresponds to the given <112> slip system of the matrix.) Until this initiation point, the twin orientation was co-rotated with the mother grain. The co-rotation was stopped as soon as the twin had at least 1% volume of the mother grain. Starting from that point, the lattice rotation of the twin is dictated by its own slip system activity, which is different from its mother grain, due to the different orientation of the twin.

Except for a direct reorientation of the twinned volume, twinning also influences the texture evolution due to a strong latent hardening effect [12,47,48]. The twins are assumed to be harder than the parent grains [49,50,51], which was considered in the present modeling by assigning higher hardening rate for the twins with respect to the matrix (see the hardening approach in Section 4.3). Furthermore, twins observed in TWIP steels appear in stacks of thin, nanoscale lamellae with small thickness (e.g., Figure 5). Therefore, enhancement of the twin volume fraction is dominated by the nucleation of new lamellar structures rather than by the expansion of previously existing ones [52].

Due to their large volume fraction—even if they are hard—the twins must deform plastically due to the large imposed strain. This induces the formation of new twins, i.e., secondary twins are formed inside primary twins. This behavior has been observed frequently in TWIP steels subjected to large strains and/or complex deformation conditions, e.g., during tension [53], cold rolling [13,54], asymmetric rolling [55], and ECAP [25,56]. The present modeling approach also includes secondary twining, treating it in the same manner as primary twining. However, secondary twinning was considered only from the second pass on, as the hardening due to the first pass and the rotation during pass one and two are likely to result in activation of additional twinning systems during the second pass. All secondary variants were assigned as grains at zero strain (of the second pass), initially with zero volume fraction. The VPSC code requires to assign also elliptical shape tensors for considering their interaction with the equivalent homogeneous medium. Initially, spherical shapes were assigned to the parent grains while the twins were approximated by flat disks with an aspect ratio of 5, and were oriented parallel to the twinning plane. The shape was allowed to change with strain according to the operation mode of the self-consistent code.

### 4.3. Strain Hardening Model

Strain hardening was simulated using the self and latent hardening approach proposed by Kalidindi et al. [57] and Zhou et al. [58]. In that approach, the reference resolved shear strength of a slip system $\tau_{0}^{\alpha }$ is controlled by the following relation:

(2) $${\dot{\tau }}_{0}^{\alpha }=\sum_{\beta }H^{\alpha \beta }\left|{\dot{\gamma }}^{\beta }\right|,\alpha ,\beta =1\ldots 12$$

Here, *α* and *β* are the indices of the slip systems, ${\dot{\gamma }}^{\beta }$ is the slip rate and $H^{\alpha \beta }$ is the hardening matrix. The slip rate is calculated from the constitutive law proposed by Hutchinson [49] for strain rate sensitive slip:

(3) $${\dot{\gamma }}^{\beta }={\dot{\gamma }}_{0}^{\beta }\mathrm{sign}(\tau^{\beta }){\left|\frac{\tau^{\beta }}{\tau_{0}^{\beta }}\right|}^{1/m}.$$

Here, *τ^β^* is the resolved shear stress and m is the strain rate sensitivity index. The H*^αβ^* hardening matrix in Equation (2) is constructed from a slip system interaction matrix q*^αβ^*:

(4) $$H^{\alpha \beta }=q^{\alpha \beta }h_{0}{\left\{1-\left(\tau_{0}^{\alpha }/\tau_{\mathrm{sat}}^{}\right)\right\}}^{a}.$$

Here, $\tau_{0}^{\alpha }$ is the actual slip strength, $\tau_{sat}$ is the saturation value, h_0_ controls the hardening rate, and ‘a’ is a parameter. In the q*^αβ^* interaction matrix only four cases are distinguished [58]; collinear (q_1_), coplanar (q_2_), perpendicular (q_3_), and other slip orientations (q_4_) between two slip systems *α* and *β*. They are represented by four parameters; q_1_ = 1, q_2_ = 1.2, q_3_ = 2, and q_4_ = 1.5, respectively. Based on their definition, there is a physically justified order for three latent hardening parameters: q_1_ < q_2_ < q_3_. The fourth parameter is not defined by a specific physical argument, so it was approximated by the average value of the other three (‘all other cases’). The 12 {111}<110> slip systems and the 12 {111}<112> pseudo-slip systems for twinning were used. Suitable hardening parameter values were found by fitting to the experimental tensile hardening curve (Figure 3). An iteration procedure led to the following common values for the matrix and the twins: $\tau_{0}^{\langle 110\rangle }=\tau_{0}^{\langle 112\rangle }=167\;\mathrm{MPa}$, $\tau_{\mathrm{sat}}=1650\;\mathrm{MPa}$, and *α* = 0.5. The parameter for the hardening rate was different: $h_{0}^{matrix}=163.5\;\mathrm{MPa}$, $h_{0}^{primary\;twin}=327\;\mathrm{MPa}$, and $h_{0}^{secondary\;twin}=489\;\mathrm{MPa}$. After 0.4 tensile strain in RD, the twinned volume fraction was 0.2, which agrees well with the estimated experimental value. The experimental stress-strain curve was well reproduced, see Figure 3.

### 4.4. ECAP Texture Modeling Conditions

The simple shear model of ECAP was used to define the deformation gradient at each deformation step, with a 90° die, up to a total shear of γ=2 on the 45° plane. The simulation was advanced in 40 deformation steps for one ECAP pass with increments of Δγ=0.05, applying a macroscopic shear rate of $\dot{\gamma }=1/s$. 500 initial parent grain orientations were generated from the initial texture; each of them was completed with the 12 primary twin variant grain orientations with zero volume fractions. In this way, the total number of grain orientations was 500 + (500 × 12) = 6500. The strain rate sensitivity exponent was taken to 0.166 in the viscoplastic constitutive law in Equation (3). This value may seem to be relatively high; however, it can be justified when the material is heavily deformed. Namely, it is expected that the yield surface becomes more and more rounded when the dislocation density increases because individual dislocations have different critical resolved shear stresses due to their different curvatures and interactions with other dislocations. While the plastic response of a complicated dislocation structure cannot be modelled, the rounded shape of the yield surface can be considered by using higher m values. The rounding effect of the m parameter on the yield surface was shown first in Ref. [44]. It was also found in the present simulations that higher slip viscosity led to better relative strengths of the texture components. The modeling parameters are collected in Table 5.

## 5. Modeling Results and Discussion

In order to find the best agreement with the experimental textures, the ECAP texture simulation was carried out by iteration. The parameters to fit were the *α* parameter in the localization equation of the VPSC model [38,39] and the strain rate sensitivity index. The hardening parameters were not varied again, because they were fitted to the tensile strain hardening curve (Figure 3). By varying the *α* parameter in the VPSC model, one can continuously change the nature of the interaction between a grain and the homogeneous equivalent medium around the grain, which represents the polycrystal. *α* = 0 corresponds to the Static model, where the stress state is the same for all grains, while *α* = ∞ is the Taylor model with uniform strain. Best texture results were obtained by taking the *α* parameter to 100, which is equivalent to the Taylor model in practice. Concerning the strain rate sensitivity of slip, the m = 0.166 value was adopted.

**Figure 6:**
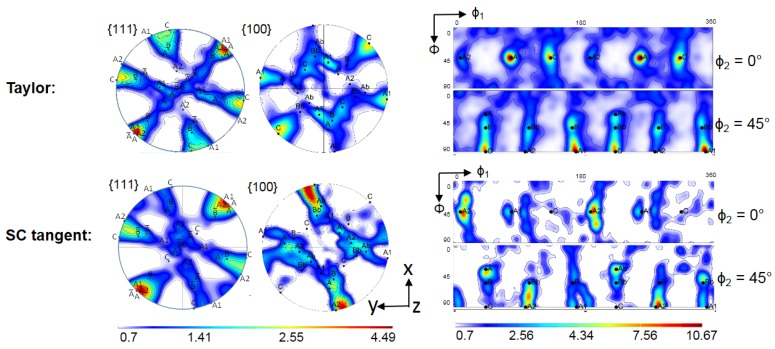
Simulated textures after one-pass ECAP obtained by the Taylor and the self-consistent (SC) (tangent) model in {111} and {100} pole figures, as well as in the φ_2_ = 0° and 45° ODF sections.

**Figure 7:**
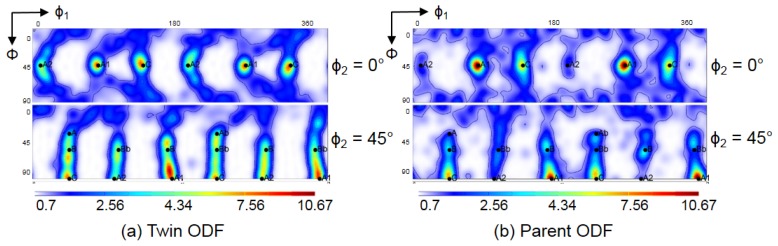
Decomposition of the full, simulated texture obtained by the Taylor model in Figure 6 into (**a**) primary twins only and (**b**) parent grains only. The textures are presented by φ_2_ = 0° and 45° ODF sections.

**Figure 8:**
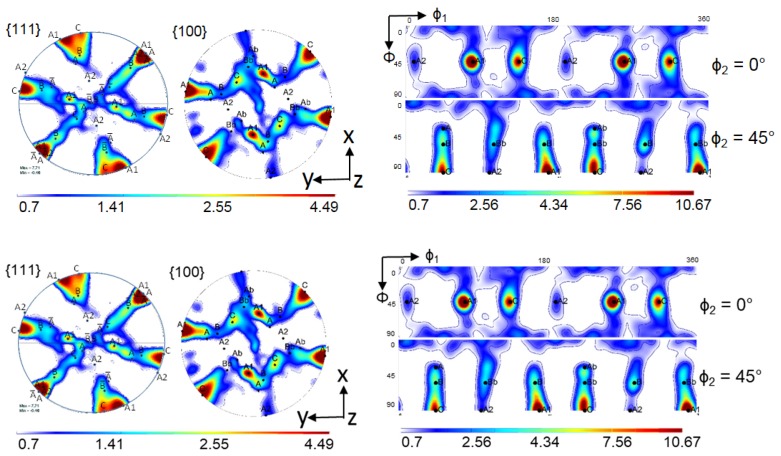
Simulated texture after one-pass ECAP obtained by the Taylor model without twinning in {111} and {100} pole figures as well as in the φ_2_ = 0° and 45° ODF sections.

**Figure 9:**
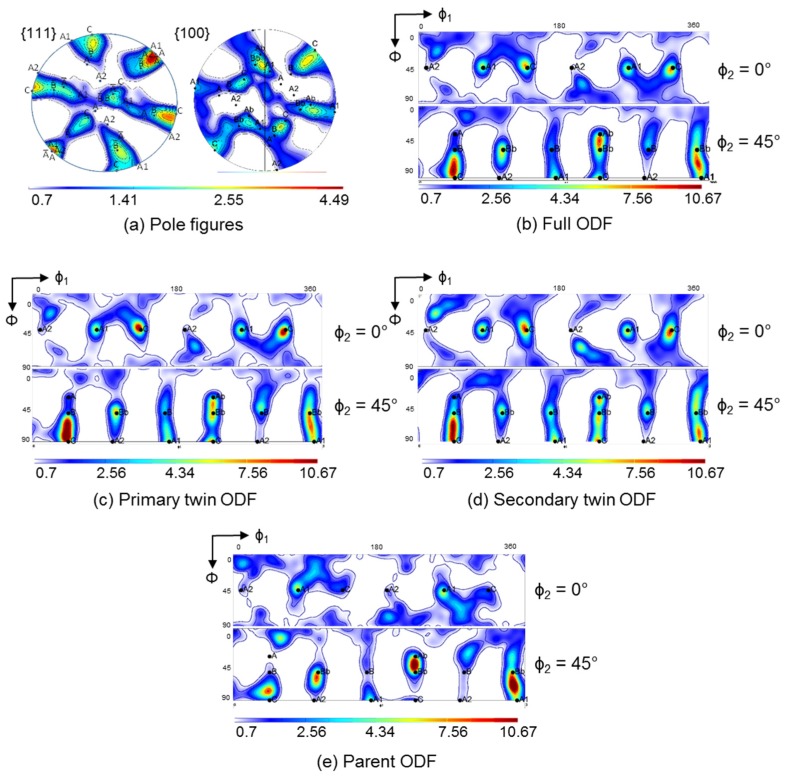
Simulated texture obtained by the Taylor model after two-pass ECAP. (**a**) {111} and {100} pole figures; (**b**) ODF of all grains; (**c**) ODF of primary twins only; (**d**) ODF of secondary twins only; and (**e**) ODF of parent grains only. ODFs are given as φ_2_ = 0° (top) and 45° (bottom) sections.

The simulation results for the crystallographic textures after the first and second pass are presented in Figure 6, Figure 7, Figure 8 and Figure 9, respectively. In general, a very good agreement between experimental and simulated textures was achieved, when using the Taylor model and taking mechanical twinning into account. Detailed discussion of the texture evolution is presented in the following Section 5.1. For a better comparison with the experimental textures (Figure 2), the same intensity levels were used in the simulated textures. For the construction of the ODFs, the Gaussian spread around the grain orientations was 7° and the harmonic coefficients were calculated up to the rank of 32.

### 5.1. Texture Evolution

**Figure 10:**
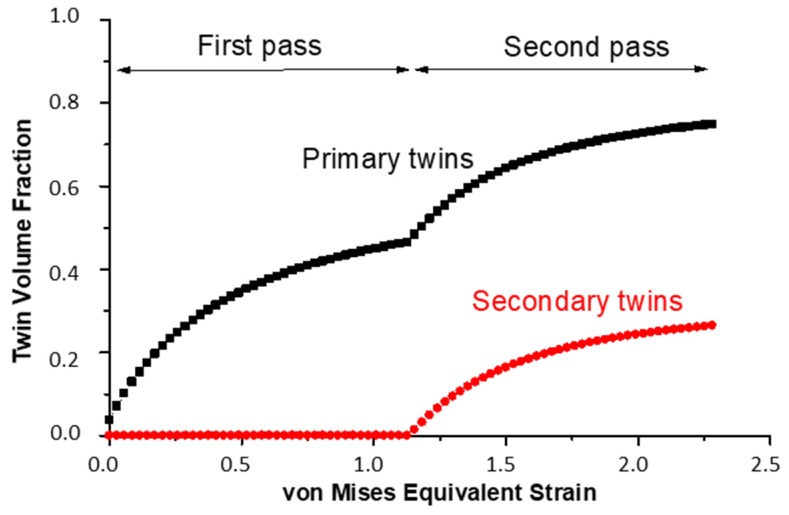
Simulated evolution of the twin volume fraction as a function of the von Mises equivalent strain during the first and second pass. Secondary twins were activated only from the start of the second pass.

As can be seen from a comparison between the experimental (Figure 2) and simulated results (Figure 6 (Taylor) and Figure 9), the texture intensities are very similar and the relative strengths of the main texture components was well predicted. The first point of discussion is the meaning of the Taylor deformation mode that was identified by the simulation. This is quite unusual, because previous modeling of fcc polycrystals subjected to large deformation showed low *α* values [35]. In order to verify the validity of the Taylor model, a calculation of the first ECAP pass using the same parameters, except for the *α* parameter, was performed with the tangent approach of the self-consistent (SC) model, i.e., with *α* = m = 0.166. The obtained results are shown in Figure 6. The Taylor model correctly predicted weaker A, Ab and A2, intermediate B and Bb, and stronger A1 and C texture components. In contrast, the SC model overestimated the A and A2 texture components, whereas the main experimental components A1, B, and C were underestimated. The relative strengths of the texture components were also calculated incorrectly by the SC model. The applicability of the Taylor model must come from the high twinning activity. Indeed, the predicted volume fraction of primary twins was 46% after the first pass, and increased up to 74% during the second one. During the second pass, there was also secondary twinning, occupying 26% of the total volume, see Figure 10. They were, however, included into the primary twins, so secondary twinning did not decrease the parent grain volume fraction, which is then also 100% − 74% = 26% after the second pass.

The obtained large twinning volume fractions at the end of the first (46%) and second pass (74%) seem to be comparatively high. Indeed, observations by EBSD indicate a lower volume fraction. This difference needs explanation. First of all, it is emphasized that the capability of the EBSD technique to resolve deformation twins with nanoscale width and separation distance quantitatively is highly limited, which leads to an underestimation of the volume fraction, and so does not permit exact quantitative analysis. In addition, EBSD measurements can only capture twins that are near twinning positions and are unable to detect twins that were created before but rotated away from their twin orientation. Those twins can only be recognized from their morphology, that is, by their lamella-type shape. This is why we conducted the analysis presented in Figure 4 and in Table 3 and Table 4. Therefore, the simulation result can be reasonable, meaning that only one third of the total twins remained near their twin orientation.

The twins take the form of lamellae and occupy 74% of the volume. They are also very hard (see Section 4.2) and interconnected. According to the microstructure observations, the twins constitute a skeleton that fills up the whole volume of the parent grain. When such a structure is subjected to large plastic deformation, the hard twin skeleton, with its large volume fraction, enforces uniformity of plastic strain in the whole grain. A consequence of the strain homogeneity within individual grains is that all grains deform in the same way. This conclusion comes from the fact that strain heterogeneity between adjacent grains of the polycrystal must be accommodated near the grain boundary regions of individual grains, which is different for each neighbor. This difference is not possible because the strain is the same everywhere in a given grain. Therefore, the homogeneity of strain within each grain of the polycrystal induces a homogeneous deformation of the whole polycrystal, that is, the Taylor deformation mode.

Our investigation led to the fundamental result that the polycrystal is deforming according to the Taylor model, which is a uniform deformation. Therefore, the twins deform the same way as the imposed macroscopic deformation, with the same strain mode as the mother grains. In this way, compatibility is maintained between the twins and the mother grains. Normal slip was deforming the twins when they were formed. Secondary twinning was allowed only at large strains, starting from the second ECAP pass, which was also taking place according to the Taylor model.

### 5.2. Effect of Twinning on Texture Evolution

**Figure 11:**
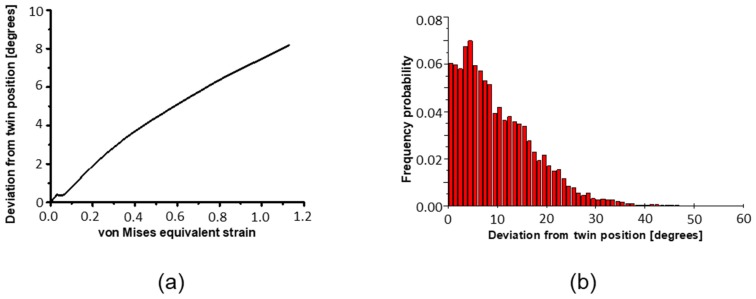
(**a**) Average deviation from exact twin position during the first ECAP pass; (**b**) Histogram showing the distribution of the simulated deviation. (Bin size: 1°, largest deviation: 48°.).

It is important to emphasize that the present modeling is for large strains, up to a shear of *γ* = 4, during which the initial twins can rotate significantly and differently with respect to their parent grains, according to their own slip system activity. This effect was considered in the present simulation according to the scheme described in Section 4 above and proven experimentally, as shown in Figure 4 and Table 4. Indeed, by the end of the first ECAP pass, the average disorientation from the exact twinning position had increased by up to 8.2°, as shown by the distribution of the disorientations in Figure 11a,b. The disorientation achieves values of up to 48°, which is similar to the maximum value observed experimentally (50°). Therefore, it was important to consider these deviations for controlling the transfer of volume between parent grains and twin variants. Indeed, to account for the increase in deviation, the volume transfer was progressively reduced, and finally stopped for disorientations larger than 10°. After 10° deviation, a twin is no longer a twin, but a normal grain, which follows its own orientation change without further growth. This is why the average rate of volume transfer is progressively reduced with the strain, and only a fraction of twins will continue growing after the first pass (Figure 11b).

In order to determine the influence of deformation twinning on texture evolution, the simulation of the first ECAP pass was also performed using the Taylor model, with the same simulation parameters, but without taking twinning into account (Figure 8). Comparison of the texture with and without twinning (Figure 6 and Figure 8) evidences that when deformation twinning is not considered as an additional deformation mechanism, the simulated texture deviates strongly from the experimental one. For instance, the A1 and C texture components are strongly overestimated, whereas the B and Bb texture components were not predicted as separate components at the orientation positions (165°/54.74°/45°) and (345°/54.74°/45°), respectively, without twinning. This comparison also shows that deformation twinning leads to strong decrease in the general intensity of the texture, i.e., twinning has a randomization effect. Twins are in different positions with respect to their parent grains, so if the parent grain is near an ideal orientation, the twin cannot approach the same one. This is one of the reasons why the intensity of the simulated texture (Taylor model including twinning) approaches the experimental one. This effect was also observed in quantitative grain fragmentation simulations [50], where the new grains developed orientations that differed from those of their parents.

In order to identify the effect of twinning on the texture evolution, the textures that correspond only to the twin and parent orientations are also plotted separately in Figure 7 and Figure 9. At first, they appear quite similar to each other. Close inspection, however, shows the following major differences: (i) the texture intensity is significantly lower for the twins compared to that in the parent grains; (ii) systematically, the A2 component is very weak for the parent grains; (iii) the A, B, Ab, and Bb texture components are more pronounced in the twin ODF; (iv) for the second pass, where secondary twinning was activated, the A2 and the B component of the ideal texture components of the parent grains are also absent. At the same time, the strong intensity between the B and C components observed experimentally was also well simulated by the primary and secondary twin ODFs.

**Figure 12:**
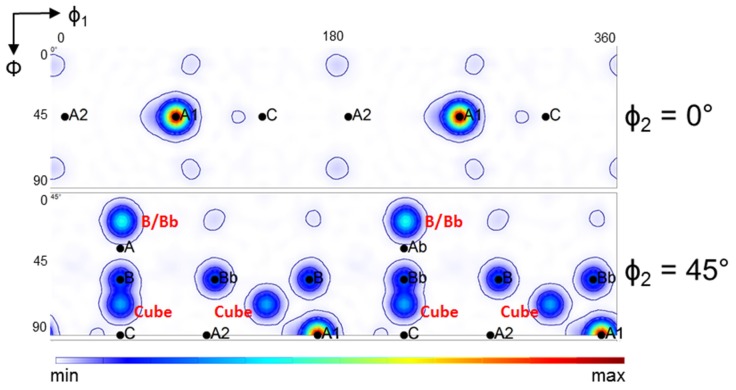
The twin positions and their relative strength formed by the ideal texture components in ECAP, taking all ideal texture components with the same intensity. Ideal orientations twinning into each other are indicated with black letters: the A1 is originating from A2, the B is from Bb, inversely, the Bb from B. The Cube-originated twins are indicated by red letters, also two twin components of B/Bb that are not in ideal positions for simple shear.

**Figure 13:**
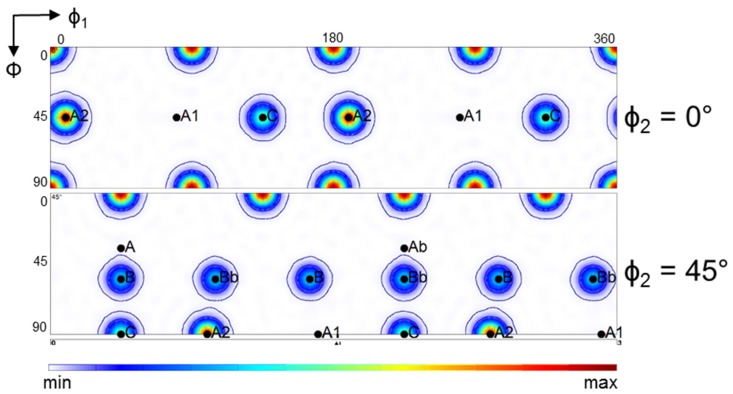
The relative twinning capacity of the ideal ECAP texture components and of the Cube orientation. The Cube orientation is the most prone to twin, followed by the A2, C and the B/Bb components. A1 and A/Ab do not twin.

These texture features can be understood more easily if we study the twinning behavior of the ideal orientations. Figure 12 (The two-fold symmetry operation was applied for the generation of the ODFs in Figure 12, which is applicable for simple shear if the initial texture also displays that symmetry. This symmetry can be applied for the first ECAP pass, but not for the second, because the sample was rotated around its long axis by 90° for the second pass following the Route B_C_ of ECAP testing. Consequently, the two-fold symmetry was lost. And Figure 13 show the results of an analysis carried out for the twinning behavior of ideal orientations of simple shear textures (here ECAP). For the construction of these figures, the same volume was allocated to the ideal orientations. Afterwards, the twin variants were calculated with the same approach as for the whole polycrystal. The Cube orientation was added to the analysis as it is frequently a strong component in initial textures of fcc polycrystals, as in the present case. Figure 12 displays the generated twin orientations from the ideal ones for ECAP deformation; that is, for simple shear along the 45° oriented intersection plane of the two channels. Except for the Cube orientation, all generated twin orientations lie in the φ_2_ = 0° and 45° sections (for the Cube, there are also twins in the φ_2_ = 27° section of Euler space). The result of the theoretical analysis shows that the A2 texture component twins into A1. Also, there are no twins generated into the A2, C, A and Ab positions. The B/Bb orientations twin into each other, and also into a new component, as indicated by the red color in Figure 12. The Cube texture component twins into stable shear-texture components lying on the so-called B-fiber of shear textures; positioned between the C and B, as well between the C and Bb orientations in the φ_2_ = 45° ODF section (Figure 12). The other twin variants of the Cube orientation do not appear in the ideal positions of shear. The relative intensities of the twin orientations are also indicated with the intensity levels around them in Figure 12. It is clear that the A1 component is the strongest twin orientation, followed by the B/Bb twins.

Another way to study the twinning behavior of the ideal orientations is to plot the twinning capacity of the ideal orientations. This is the inverse of the analysis shown in Figure 12. As shown in Figure 13, the Cube texture component is the most prone to twin, followed by the A2, the C and the B/Bb texture components. The A1 and A/Ab orientations do not twin.

On the basis of the twinning behavior analyzed theoretically in Figure 12 and Figure 13, the following interpretation of the experimentally observed texture evolution can be made:

- The A2 texture component is very weak, because it twins into A1, which becomes the strongest texture component after the first ECAP pass and remains strong after the second pass. Due to the high fraction of twinned volume, weakening of the A2 texture component is further facilitated with increasing strain, i.e., the intensity of A2 decreases after the second pass. These findings are in agreement with observations made in a previous study on silver, which has a low SFE and shows severe deformation twinning, deformed by ECAP (four passes, route A) [51].
- The B/Bb texture components are relatively strong because they are ideal shear texture components and because they twin into each other. Two new components can be formed by the B/Bb twins (indicated in red in Figure 12), which are not ideal components of simple shear textures. They are present in the experimental texture after the first pass, though they are quite weak. The existence of these components can only be explained with a co-rotation that may take place between the ideal B/Bb parent grain and its embedded twin variant in the new orientation. Such lattice co-rotation would require the same slip system activity in the twin as in the parent grain, which is unlikely, as there are more slip systems active in those new positions than in the ideal B/Bb orientations (see in Ref. [59]). Therefore, the two observed slight components are probably only temporary variations of the texture intensity.
- Primary twins can increase the strengths of all ideal shear-texture components in the first pass (see Figure 7a), because they rotate out of their twinning positions during large strain (Figure 11). The twins can finally reach an ideal position by their own slip activity. This effect can be seen as the reason for the strengthening of the A, B, Ab, and Bb texture components, which were comparatively weak in the simulated parent ODF, but present in the twin-ODF and in the experimental ODF.
- Cube-oriented grains, which dominated the initial hot-rolled texture, twin heavily and can be responsible for the high ODF intensities between the C-B and C-Bb orientations in the first pass. In the second pass, only the orientations between C and B remain strong. The latter is due to the sample rotation between the first and second ECAP pass in Route B_C_, which destroys the two-fold symmetry of the texture and impedes the formation of the texture component between C and Bb.

Secondary twinning must be operational during the second ECAP pass, because the experimental texture can only be reproduced if secondary twinning is introduced into the simulation. While secondary twinning might also take place during the first pass, this was not observed experimentally and therefore not modelled. There is no dramatic difference between the ODFs formed by the primary and the secondary twins (Figure 9c,d), while the parent grains display a very different texture (Figure 9e). The reason for the latter is that 74% of the parent grain volumes are converted into twins, so the heavily twinning orientations disappear. More specifically, the experimental ODF maximum between the C and B texture components after two passes was well simulated by the primary and secondary twin ODFs, and the weaker intensity between the C and Bb texture components was also captured. Moreover, not all components can form in the second pass because of the loss of two-fold symmetry.

## 6. Conclusions

The texture evolution during ECAP of a high-manganese Fe-23Mn-1.5Al-0.3C TWIP steel subjected to one and two ECAP passes was analyzed. The VPSC model was updated to simulate the ECAP texture using a sophisticated volume transfer scheme to account for the effect of twinning. The results of the study provide the basis for the following main conclusions:

1. The new volume transfer scheme was proven to be a robust approach for quantitative modeling of the effect of mechanical twinning on the evolution of the crystallographic texture without employing any variant selection criterion;
2. The twin relation is progressively lost between the twins and their parent grain during large strain, for which it is necessary to control the twin growth and to finally stop after a given disorientation. This mechanism was modeled within the VPSC scheme and led to very good agreement between experimental and simulated textures;
3. Both primary and secondary twinning were accounted for in the present simulations and were found to be essential for correct calculation of the crystallographic textures. The A1, B, and Bb texture components after one pass and the orientations between C and B after two passes are directly related to the occurrence of deformation twinning in fcc alloys with low stacking fault energies;
4. The plastic deformation of the TWIP steel polycrystal was found to take place under the upper-bound Taylor conditions, i.e., under homogeneous deformation, which is due to the composite nature of the parent-twin structure imposing uniform strain within a grain and is subsequently applied to the whole polycrystal. Therefore, it was proven that self-consistent schemes can be bypassed under the conditions investigated.

## Figures and Tables

**Table 1:** Chemical composition of the investigated alloy.

Element	Fraction (wt.%)
Fe	bal.
Mn	22.46
Al	1.21
C	0.325
Si	0.041
N	0.015
P	0.01

**Table 2:** Definition of the ideal texture components for an ECAP die with 90° angle.

Texture Component	φ_1_ (°)	Φ (°)	φ_2_ (°)
A_1_	80.26/260.26	45	0
	170.26/350.26	90	45
A_2_	9.74/189.74	45	0
	99.74/279.74	90	45
C	135/315	45	0
	45/225	90	45
A	45	35.26	45
Ab	225	35.26	45
B	45/165/285	54.74	45
Bb	105/225/345	54.74	45
{111}-/A-fiber	{111}<uvw>		
<110>-/B-fiber	{hkl}<110>		

**Table 3:** The mean orientations of the grains marked in Figure 4.

Grain	Mean φ_1_ (°)	Mean Φ (°)	Mean φ_2_ (°)
1	286.45	51.28	45.76
2	44.5	45.35	46.73
3	341.6	48.14	33.77
4	43.81	49.17	33.91
5	215.52	50.33	48.11
6	285.1	50.8	52.95
7	98.82	41.62	39.14
8	279.32	45.41	38.32
9	212.38	46.61	60
10	280.54	50.03	48.95
11	203.3	44.94	68.5
12	285.8	43.34	41.47
13	352.36	43.8	25.28

**Table 4:** Orientation relationships between twin and matrix for the 13 grains shown in Figure 4.

Combination	Disorientation and Nearest Rotation Axis	Offset from Nearest Rotation Axis (°)	Deviation from Twinning Position (°) (from 60°<111>)
1–2	19.07°<3-20>	6.21	50
2–3	53.13°<-14-3>	2.47	42.45
3–4	55.07°<423>	2.57	35.54
4–5	56.77°<-2-3-3>	2.39	10.8
5–6	48.29°<443>	1.87	15.6
6–7	50.7°<-1-4-4>	0.47	22.5
7–8	55.98°<24-3>	1.98	18.3
8–9	48.29°<433>	3.42	8.87
9–10	58.03°<-3-2-3>	3.87	10.72
10–11	58.13°<-101>	4.14	28.14
11–12	55.84°<343>	1.76	9.94
12–13	56.81°<-3-4-4>	2.52	9.7

**Table 5:** List of the polycrystal modeling parameters used.

Parameter	Value
Slip systems and initial strength, $\tau_{0}$	{111}<110>, 167 MPa
Twinning systems and initial strength, $\tau_{0}$	{112}<110>, 167 MPa
Hardening rate for mother grain, $h_{0}$	163.5 MPa
Hardening rate for primary twin, $h_{0}$	327 MPa
Hardening rate for secondary twin, $h_{0}$	489 MPa
Saturation stress, $\tau_{\mathrm{sat}}$	1650 MPa
Strain hardening parameter, a	0.5
Latent hardening parameters	q_1_ = 1, q_2_ = 1.2, q_3_ = 2, q_4_ = 1.5
Strain rate sensitivity parameter for slip and twinning, m	0.166
Interaction coefficient for Tangent VPSC model, *α*	0.166
Initial number of grains (mothers + 12 primary twins)	6500
Initial grain shape axis ratios for twins	1:5:5
